# Simultaneous and Sequential Coexposures of *Serratia marcescens* in *Caenorhabditis elegans*

**DOI:** 10.17912/micropub.biology.002064

**Published:** 2026-04-21

**Authors:** Emily B. Smith, Jennifer D. Gresham, Andrew Huang, Levi T. Morran

**Affiliations:** 1 Population Biology, Ecology, and Evolutionary Biology Graduate Program, Emory University, Atlanta, GA, US; 2 Biology, Pennsylvania State University, State College, PA, US; 3 Biology, Emory University, Atlanta, GA, US

## Abstract

Coinfections are common in nature and include hosts simultaneously or sequentially exposed to different genotypes. We investigated the potential for simultaneous and sequential exposures to impact host fitness by exposing *Caenorhabditis elegans* hosts to different bacterial strains of *Serratia marcescens* . We found that in simultaneous exposures a highly virulent strain induced high mortality regardless of coexposure. In sequential exposures, the same highly virulent strain induced high mortality if it was the first strain introduced in a coexposure. Overall, we find host outcomes after coinfections may depend on particular parasite genotype combinations and infection timing.

**Figure 1. Estimated proportional host mortality under simultaneous and sequential coexposures:** Estimated proportion of host mortality after 48 hours of simultaneous and sequential exposures to parasite strains with 95% bootstrap confidence intervals. Color corresponds to host genotype. The left subplot of each set of data shows hosts exposed to one bacterial *Serratia* strain, while the right subplot of each set of data shows hosts exposed to multiple bacterial *Serratia* strains.

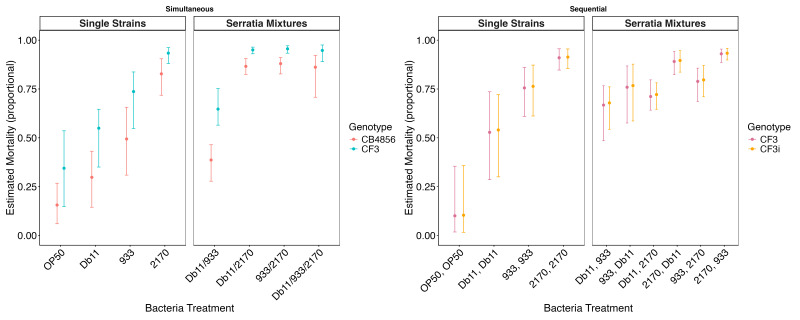



## Description

Coinfections, broadly defined as hosts experiencing two or more strain or species infections at once, are common in nature but can vary in their composition, order of exposure, duration of coexistence, and the amount of time between encounters with the initial and secondary infections (Petney and Andrews 1998; Cox 2001; Read and Taylor 2001; Fleming et al. 2006; Balmer and Tanner 2011; Rellstab et al. 2011; Thumbi et al. 2014; Rynkiewicz et al. 2015; Karvonen et al. 2019; Devi et al. 2021). Hosts can experience coinfections that are introduced at the same time (“simultaneous” coinfections), like the transmission of *Borellia burgdorferi* , the causative agent of Lyme disease, and *Anaplasma phagocytophilum* , the causative agent of human granulocytic anaplasmosis, in the bite of a single tick to a human host (Horowitz et al. 2013) or through trophic movement from a coinfected prey to a predator. Alternatively, hosts can experience coinfections that co-occur for some amount of time but are not introduced at the same time (“sequential” coinfections) such as influenza patients that secondarily experience respiratory system colonization with bacterial species that lead to pneumonia (Pasman 2012) or tomato plants infected with an avirulent strain of powdery mildew that then suppresses a secondary virulent strain (Seifi et al. 2012). The timing as well as the nature of the infecting parasites can have important consequences on host fitness, and ultimately, the evolutionary trajectories of the host and parasite populations (Telfer et al. 2008; Alizon et al. 2013; Karvonen et al. 2019; Zilio and Koella 2020; Schmid-Hempel 2021). Different strains of bacterial parasites might have additive or multiplicative effects on hosts relative to single strain infections when coexposed, but the way in which those effects might occur could encompass a variety of mechanisms.

First, coinfections differ from single infections because they create an environment in which competition between the coinfecting partners may exist. Competition between coinfections have been shown to either increase (Bell et al. 2006; Ben-Ami et al. 2008) or decrease (Gower and Webster 2005) virulence in host populations, emphasizing that the particular ecological context and specific interactions between players in coinfection scenarios may be important for predicting whether within-host competition will drive overall virulence up or down (Schjorring and Koella 2003; West and Buckling 2003; Alizon 2008; Ashby and King 2017; Karvonen et al. 2019). Another way coinfecting species may impact host-parasite dynamics is through immune priming. One coinfecting species or strain may trigger a host response that protects the host from other infections, effectively priming the host immune system to better tolerate other infections (Prigot-Maurice et al. 2022). Immune responses may alternatively be focused on a less virulent infection and could actually allow a more virulent coinfecting species to increase in frequency or select for increased virulence in the less-virulent coinfecting species, or competition among coinfecting strains may be to some degree mediated by the host immune system (Råberg et al. 2006). Coinfecting species or strains may also interact indirectly and potentially facilitate each other's success. For example, mice infected with *Heligomosomoides polygyrus* , an intestinal helminth, endure damaged epithelial junctions that then allowed for increased bacterial translocation across the epithelium (Chen et al. 2005). This kind of facilitation could result in higher virulence. Therefore, more research on the impact of coinfections on hosts is needed to better understand how coinfections might interact with each other and with their hosts.

We conducted both simultaneous and sequential exposures of different strains of the bacterial species *Serratia marcescens* (strains SmDb11, Sm933, and Sm2170) in the model host system, *Caenorhabditis elegans* . Infection timing can impact how the overall dynamic of coinfection proceeds (Lello et al. 2004; Telfer et al. 2008) and by using this host-parasite system we were able to systematically probe differences in host mortality due to simultaneously or sequentially exposed coinfections. *S. marcescens* is a gram-negative bacterial parasite that occurs commonly in soil, water, composts, and is naturally encountered by *C. elegans* in the environment (Alegado et al. 2003; Kurz and Ewbank 2003). *C. elegans* are initially attracted to *S. marcescens* and eat the bacteria, after which the bacteria colonize the intestine, causing damage to the intestinal tissue and inducing host mortality (Pradel et al. 2007). We measured host mortality in populations of *C. elegans* that were subjected to either simultaneous coexposures, sequential coexposures, or single infections.

Host strains used in these experiments include the Hawaiian strain CB4856 , and two additional strains derived from this progenitor. Strain CF3-30 (CF3), which is an obligately outcrossing mutant of CB4856 that has been previously exposed to EMS mutagenesis, and CF3-30i (CF3i), which has the wild-type allele allowing for self-fertilization backcrossed back into the CF3 background. Because CB4856 exhibited reduced mortality rates in the simultaneous experiments and sequential infections were anticipated to be less virulent, we used CF3i in the sequential experiments as a better indicator of infection dynamics than CB4856 .

**Single Strain Exposures**

Our data show that hosts exposed to *S. marcescens* strains SmDb11, Sm933, and Sm2170 exhibit increasing levels of host mortality, respectively. Simultaneous exposures in single strain infections refers to hosts exposed just to one strain. Simultaneous exposures to an *Escherichia coli* OP50 control resulted in low estimated proportional host mortality relative to other treatments, simultaneous exposures to SmDb11 resulted in higher estimated proportional host mortality, hosts simultaneously exposed to Sm933 had even higher levels of estimated proportional mortality, and hosts simultaneously exposed to Sm2170 had the highest levels of estimated proportional mortality ( Figure 1, Extended Data Table 1). Estimated proportional host mortality in the single strain sequential data follows the same trend with sequential exposure to *E. coli* OP50 inducing little mortality, sequential exposure to SmDb11 inducing moderate relative mortality, Sm933 inducing higher relative mortality, and Sm2170 inducing the highest level of relative host mortality ( Figure 1, Extended Data Table 2). This trend aligns with previous work categorizing the relative virulence of these strains in *C. elegans* (Lewis et al. 2022). In the simultaneous data, host genotype had a significant effect on mortality, with CF3-30 exhibiting significantly higher mortality than CB4856 across treatments ( Figure 1, Extended Data Table 3). There was no significant effect of genotype in the sequential single strain data.

**Multi-strain Exposures**

Simultaneous exposures including the most virulent strain, Sm2170, led to consistently high levels of relative host mortality regardless of coexposed strain identity ( Figure 1, Extended Data Table 1). This *Sm2170* precedence was supported in the sequential exposures when Sm2170 was the first of the coexposed strains in the sequence ( Figure 1, Extended Data Table 2). The relative mortality of sequential coexposures that introduced Sm2170 first hovered around roughly the same mortality range as Sm2170 in the single strain context, whereas coexposures in which Sm2170 was the secondary exposure had lower mortality ( Figure 1, Extended Data Table 2). Coexposures of SmDb11 and Sm933 generated host mortality rates that were not statistically different than either single strain ( Figure 1, Extended Data Table 4).

There are several possible explanations for why sequential exposures follow the same pattern as simultaneous exposures when Sm2170 is first in the sequence of exposures, but not the second. Strains in simultaneous exposures, while introduced at equal proportions, may end up with an over-representation of the more virulent strain after they have been in direct competition for some amount of time. Alternatively, exposing hosts to a sequence of strains may allow hosts to generate some level of tolerance or mount an immune response as a result of the first exposure before the introduction of a secondary exposure. It may also be the case that a lag in the time of parasite introduction could change the dynamic with which coexisting strains may interact with each other. A 24 hour lead time may also provide an otherwise out-competed strain to have a better chance of establishing and competing with a more virulent strain for longer. Further, the introduction of a highly virulent strain for 24 hours may provide an opportunity for that strain to dominate the gut environment before a second strain is introduced. This may explain why sequential coexposures in which hosts saw Sm2170 first had higher mortalities that were very similar to hosts exposed to single infections of Sm2170. Overall, we see no clear additive or multiplicative effects of coexposed strains on host mortality. Instead, our data, at least in the case of the most virulent bacterial strain, are consistent with simple competitive dominance hierarchy among strains that may determine the virulence of the interaction with the host. It's plausible that bacterial virulence may be directly associated with competitive ability or growth rate inside the host, thus allowing Sm2170 to be the dominant strain. Nonetheless, it appears that priority effects, or exposure order, also play a role in determining the outcome of these coexposures. These results were consistent across host genotypes CF3-30 and CF3-30i, but other host genotypes may vary in their response.

Overall, we conclude that the effect of coexposures on estimated proportional host mortality does not have a clear additive or multiplicative effect in the *S. marcescens* / *C. elegans* system, but priority effects, or exposure order, may influence virulence outcomes. Therefore, particular strain interactions in addition to timing of exposure may be important for determining the outcome of coexposures.&nbsp;

## Methods

**Nematode Maintenance and Bacterial Strains**

The *C. elegans* strain used for these experiments were all variants of the Hawaiian strain CB4856 . The CF3-30 strain (referred to in Figure 1 as CF3) was created previously (Morran et al. 2009, 2011) from CB4856 *C. elegans* which contain the *fog-2 ( q71 )* mutant allele which prevents spermatogenesis in hermaphrodites (Schedl and Kimble 1988) making them obligately outcrossing. The CF3-30i strain (referred to in Figure 1 as CF3i) was created from backcrossing the wild-type *fog-2 ( q71 )* allele back into the CF3-30 background, restoring hermaphrodite spermatogenesis function. These populations were maintained on 100mm Petri dishes filled with Nematode Growth Medium-Lite (NGM-Lite, US Biological, Swampscott, MA, USA) and seeded with 200µl of *Escherichia coli* OP50 ( Caenorhabditis Genetics Center, University of Minnesota). Nematodes were allowed to grow, feed, and reproduce on these plates and were stored at 20°C.

The strains of *S. marcescens* and E. coli OP50 used for these experiments were grown in Luria Broth to carrying capacity, shaking overnight at 28°C. These liquid cultures were then used to seed Serratia Selection Plates (SSPs) as described previously (Morran et al. 2011). Strains used for both simultaneous and sequential infections included Sm2170 (Sue Katz, Rogers State University), Sm933 (Carolina Biological Supply), and SmDb11 ( Caenorhabditis Genetics Center, University of Minnesota). The *C. elegans* hosts show signs of infection with *S. marcescens* 24 hours after infection and interactions between *S.marcescens* and *C. elegans* have been previously characterized (Schulenburg and Ewbank 2004).

**Exposure Assays**

Serratia Selection Plates (SSPs), as described previously (Morran et al. 2011), were used to expose hosts to parasite strains. Briefly, the left third of the plate was seeded with the parasite strain of choice, the right third of the plate was seeded with *E. coli* OP50 food, and the middle third was not seeded and contained a strip of Ampicillin to prevent the carry-over of the parasite into the food lawn. The worm hosts were placed on the parasite lawns at the start of the assay and were given 24 hours to interact with the parasite and crawl across the plate to the lawn of food. After the first 24 hours, the surviving worms on the lawn of food were liquid transferred with M9 to another SSP for another 24 hours. The number of worms alive on the lawn of food after the second SSP exposure was counted after a total of 48 hours of potential parasite exposure. Simultaneously infected hosts were exposed to proportionally equal mixtures of bacterial strains and moved to another replicate of the same mixture to control for movement of worms after 24 hours. Sequential exposures were conducted by exposing host populations to a single bacterial strain lawn for 24 hours and then surviving individuals were moved to a secondary bacterial lawn for another 24 hours before survival counts were collected. Simultaneous assays were run in triplicate with 200 worms per replicate population. Sequential assays were conducted with 300 worms per population and five replicates per treatment.

**Statistical Analysis**

Mortality was analyzed as a continuous proportion using generalized additive models (GAMLSS) implemented in the *gamlss* package in R (Rigby and Stasinopoulos 2005). Because the mortality data included both exact zeros and ones, we fit a zero- and one- inflated beta distribution (BEINF), which explicitly models the probability of observing structural zeros and ones in addition to variation in intermediate values. Treatment and host genotype were included as fixed effects on the mean parameter (μ), while scale (σ) was modeled as constant, and the zero/one inflation parameter (ν) was allowed to vary with both treatment and genotype to account for potential heterogeneity in the frequency of zeros/ones across experimental conditions. Model adequacy was assessed using randomized quantile residuals and visual inspection of fitted values, which indicated good model convergence and adequate fit. Uncertainty in predicted mortality estimates were quantified using nonparametric bootstrapping of the BEINF model (n = 1,000, resampling with replacement, random set seed for reproducibility). Predicted means and 95% confidence intervals were calculated from the resulting bootstrap distributions. While the model was fit on the logit scale, all reported values and confidence intervals are back-transformed to the original 0–1 scale for interpretability and consistency with the figures. Pairwise comparisons between all treatments were then conducted post-hoc (Extended Data Table 4, Extended Data Table 6). All analyses were conducted in RStudio (RStudio Version 2025.05.1+513).

## Data Availability

Description: Data Tables. Resource Type: Dataset. DOI: https://doi.org/10.22002/q3f11-wwv74
